# Vascular endothelial growth factor regulates osteoblast survival – evidence for an autocrine feedback mechanism

**DOI:** 10.1186/1749-799X-4-19

**Published:** 2009-06-16

**Authors:** John Street, Brian Lenehan

**Affiliations:** 1 Department of Orthopedic Surgery, National University of Ireland, Cork, Ireland; 2 Combined Neurosurgical and Orthopedic Spine Program, University of British Columbia, Vancouver, BC, Canada

## Abstract

**Background:**

Apoptosis of osteoblasts and osteoclasts regulates bone homeostasis. Skeletal injury in humans results in 'angiogenic' responses primarily mediated by vascular endothelial growth factor(VEGF), a protein essential for bone repair in animal models. Osteoblasts release VEGF in response to a number of stimuli and express receptors for VEGF in a differentiation dependent manner. This study investigates the putative role of VEGF in regulating the lifespan of primary human osteoblasts(PHOB) in vitro.

**Methods:**

PHOB were examined for VEGF receptors. Cultures were supplemented with VEGF(0–50 ng/mL), a neutralising antibody to VEGF, mAB VEGF(0.3 ug/mL) and Placental Growth Factor (PlGF), an Flt-1 receptor-specific VEGF ligand(0–100 ng/mL) to examine their effects on mineralised nodule assay, alkaline phosphatase assay and apoptosis.. The role of the VEGF specific antiapoptotic gene target BCl2 in apoptosis was determined.

**Results:**

PHOB expressed functional VEGF receptors. VEGF 10 and 25 ng/mL increased nodule formation 2.3- and 3.16-fold and alkaline phosphatase release 2.6 and 4.1-fold respectively while 0.3 ug/mL of mAB VEGF resulted in approx 40% reductions in both. PlGF 50 ng/mL had greater effects on alkaline phosphatase release (103% increase) than on nodule formation (57% increase). 10 ng/mL of VEGF inhibited spontaneous and pathological apoptosis by 83.6% and 71% respectively, while PlGF had no significant effect. Pretreatment with mAB VEGF, in the absence of exogenous VEGF resulted in a significant increase in apoptosis (14 vs 3%). VEGF 10 ng/mL increased BCl2 expression 4 fold while mAB VEGF decreased it by over 50%.

**Conclusion:**

VEGF is a potent regulator of osteoblast life-span in vitro. This autocrine feedback regulates survival of these cells, mediated via a non flt-1 receptor mechanism and expression of BCl2 antiapoptotic gene.

## Introduction

Bone is a complex, dynamic and highly specialized tissue that undergoes continuous regeneration and remodeling throughout life. Deposition and resorption of mineralized matrix occurs during development and growth, during physiological adult skeletal remodeling and during repair of surgically or traumatically injured bone. Appropriate blood supply, and intricate coupling of the vasculature with osteoblasts and osteoclasts is a prerequisite to regulation of this formation and removal of bone. Blood vessel formation, angiogenesis, and blood vessel removal, pruning, are strictly coordinated to facilitate the ever-changing demands of the skeleton. Within the temporary functioning structure of the basic multicellular unit (BMU), osteoblasts mediate bone formation, osteoclasts bone resorption, while both cells share intimate proximity with the vascular endothelium and haemopoietic and stromal cells of the bone marrow. These BMU's represent the spatial and temporal orchestration of the strictly controlled activities of osteoblasts, osteoclasts and cells of the vascular tree. The function of these cells is regulated by a number of systemic and local factors that modulate bone metabolism and vasclarization [1]. The systemic factors include parathyroid hormone, growth hormone, Vitamin D3, glucocorticoids, calcitonin and numerous vasoactive peptides. Local soluble factors known to enhance the formation of mineralized matrix include the insulin-like growth factors (IGF-I and -II), transforming growth factor beta (TGFβ), platelet derived growth factor (PDGF) and basic fibroblast growth factor (bFGF). Cytokines that enhance osteoclast function and bone resorption include interleukin-1 (IL-1), interleukin-6 (IL-6) and tumor necrosis factor alpha (TNFα) [2]. The principle 'angiogenic' cytokines that regulate blood vessel formation are vascular endothelial growth factor (VEGF), bFGF, PDGF, TGFβ, TNFα and angiopoietin-1 (Ang-I). Clearly the activities of many of these factors are common to the regulation of bone forming, bone resorbing and endothelial cells. Of these factors, vascular endothelial growth has been the focus of most recent interest [3]. This dimeric glycoprotein, with a molecular weight range from 17 to 22 kDa, has several isoforms with very similar biological activities. For a long time, VEGF was considered endothelial cell specific, however recent reports have confirmed the presence of VEGF receptors, flt-1 and/or KDR on numerous other cell types, including osteoblasts [4]. Placenta Growth Factor is another angiogenic protein specifically of the VEGF family. This protein is known to bind to Flt-1 receptor with high affinity but fails to bind the KDR VEGF receptor [5]. Recent studies have demonstrated that the mitogenic and antiapoptotic effects of the VEGF proteins on endothelial cells are mediated through specific receptors [5]. We have reported that isolated skeletal injury in humans results in local and systemic 'angiogenic' responses primarily mediated by VEGF [6,7]. VEGF has been identified as essential for bone repair in animal models [8], and is a prerequisite to hypertrophic cartilage removal and ossification during murine skeletal growth [3,5,9]. Osteoblasts may release VEGF in response to a number of stimuli, including myriad bone derived cytokines and hypoxia, simulating bone injury [[10-15]ejost]. Osteoblasts also express receptors for VEGF in a differentiation dependent manner [4]. Meanwhile osteoclasts express VEGF receptors and osteoclast differentiation and bone resorption is enhanced by VEGF in osteopetrotic mice in the absence of macrophage colony stimulating factor (MCSF) [16]. Whether VEGF has any direct effects on osteoblast activity or life span, and which receptors may be specific for this signal transduction is unknown.

The life-span of a BMU far exceeds that of the composite cells and so continuous turnover of these cells is mandatory for skeletal homeostasis [1,2]. The average bone forming life-span of an osteoblast is 10 – 14 weeks, at which time the alternative two fates are either to become buried within the lacunae of mineralized matrix as an osteocyte, or to become an elongated lining cell on the quiescent unmineralized surface of bone. Examination of human bone reveals that approximately 65% of the osteoblasts initially present within a BMU cannot be accounted for after enumeration of lining cells and osteocytes. These cells have most likely died by apoptosis, or programmed cell death, and been rapidly phagocytosed, and are thus 'missing' [2]. Indeed apoptotic cell death of osteoclasts and osteoblasts is a key regulator of the balance between bone formation and resorption in an active BMU [17]. While the rate of osteoblast programmed cell death in active seams of normal human bone is extremely uncommon, significantly increased osteoblast and osteocyte apoptosis characterize various pathological conditions e.g. postmenopausal osteoporosis, glucocorticoid induced osteopenia, rheumatoid and septic periarticular osteoporosis and avascular necrosis [2,18-23]. Increased turnover of bone forming cells is also seen within normal fracture callus [24], a temporally dependent phenomenon which can be modulated by the exogenous administration of IL-1β and TGFβ. As each of these physiological and pathological conditions of bone are inexorably linked with alterations and perturbations in skeletal vascularization one could conceptualize at least a role for the vasculature, and for angiogenic cytokines in particular, in modulating osteoblast life-span within the BMU.

The aim of this study, therefore, was to elucidate the mechanisms of apoptosis in primary human osteoblasts and to examine the effects of numerous angiogenic cytokines on osteoblast life-span. In particular we investigate the direct effects of vascular endothelial growth factor and its Flt-1 specific mutant PlGF on osteoblast differentiation, bone formation and apoptosis.

## Methods

### Study Design

Primary human osteoblast cultures are examined for expression of VEGF receptors and subsequently supplemented with VEGF 165, a neutralising antibody to VEGF and PlGF to examine the direct mitogenic effects of these angiogenic proteins by mineralised nodule and alkaline phosphatase assays. The effects of these receptor specific VEGF family members on steoblast apoptosis is then determined and their mechanism of 'survival protection' elucidated. The role of the VEGF specific antiapoptotic gene target BCl2 is then determined by osteoblast cell transfection.

#### Primary Human Osteoblast Cultures

Primary normal human osteoblasts were cultured from trabecular bone explants obtained at the time of orthopaedic procedures performed on consenting young adults who had no evidence of metabolic bone disease. The bone fragments were washed extensively and repeatedly with culture medium to remove adherent marrow cells and to expose the trabecular surface of the bone. Small bone chips (1 × 1 × 1 mm) were then placed in culture flasks (75 cm^2^), each containing 15 mls α modified Earle's medium supplemented with 10% heat inactivated fetal calf serum, penicillin (100 U/mL), streptomycin (50 μg/mL; α MEM – 10% FCS) and cultured at 37°C in a humidified atmosphere with 5% CO_2_. Cell outgrowth from the trabecular bone surfaces was apparent after 5 days, and the osteoblast-like cells became confluent after 10 – 14 days of culture. Verification of osteoblast lineage was performed by mineralised bone nodule formation assay, alkaline phosphatase activity of the cell lysate using sodium p-nitrophenyl phosphate substrate and by FACS analysis for osteocalcin. Cell passages were performed by incubating confluent cells 0.25% trypsin diluted in calcium and magnesium free phosphate buffered saline. Experiments were performed on osteoblasts subcultured to passage 3 – 6.

#### Human osteoblast cell line culture

The primary human osteoblast cultures described above were used for n = 3 experiments, each in triplicate, for each step of the study design. Their use was limited because of the difficulty in obtaining specimens and of the technical difficulty in harvesting and culture. For the remainder of the experiments the primary human osteoblast cell line NHOst (Clonetics, San Diego, California, USA) was used. These cells have not been transformed and so have a limited lifespan in culture. Preliminary studies confirmed that there were no significant differences in activity, receptor expression, cytokine release and response to angiogenic factors between the two cultures of osteoblasts. The NHOst cell line were cultured in osteoblast basal medium (OBM™) supplemented with ascorbic acid, fetal bovine serum and gentamicin/amphotericin-B as per the manufacturers protocol. Cell passages were performed by incubating confluent cells 0.25% trypsin diluted in calcium and magnesium free phosphate buffered saline. Experiments were performed on osteoblasts subcultured to passage 3 – 6.

#### Reagents, assay kits and recombinant proteins and antibodies

All reagents were purchased from Sigma Chemical Co. (St. Louis, Missouri, USA) unless otherwise stated. Adult normal human osteoblasts (NHOst) and NHOst culture media and detatchement kits were purchased from Clonetics (Walkersville, Maryland, USA). Recombinant human proteins vascular endothelial growth factor, basic fibroblast growth factor, insulin-like growth factor-1, platelet derived growth factor, placenta growth factor and tumor necrosis factor alpha were purchased from R&D Systems (Minneapolis, Minnesota, USA). Neutralising antibody to VEGF and the isotype control antibody and the Human VEGF Biotinylated Fluorokine kit were also purchased from R&D Systems (Minneapolis, Minnesota, USA). The CD 95 ligand anti-APO-1/Fas monoclonal antibody was purchased from Boehringer Ingelheim Bioproducts Partnership (Heidelberg, Germany).

#### Analysis Of VEGF binding by Osteoblasts

Primary human osteoblasts were trypsinized from 75 mm^2 ^flasks (Falcon) and returned in round bottomed polypropelene tubes (Falcon) to 37°C for 6 hours to allow regeneration of cell surface receptors (recovery period). Cells were harvested by centrifugation at 500 × g for 5 minutes and then washed twice with PBS to remove any residual growth factors that may be present in the culture medium. Cells were resuspended in PBS to a final concentration of 4 × 10^6 ^cells/mL. 10 μL of biotinylated VEGF reagent (4.5 μg/mL) was added to 25 μL of the washed cell suspension in a 12 × 75 mm tube. As a negative control, an identical sample of cells was stained with 10 μL of biotinylated negative control reagent (soybean trypsin inhibitor at 5 μg/mL). The cells were then incubated at 4°C for 2 hours at which time 10 μL of avidin-FITC reagent was added. The cell suspension was further incubated at 4°C in the dark for 30 minutes. The cells were then washed twice with 2 mL of buffered saline-protein solution to remove unbound avidin-fluorescein and resuspended in 200 μL of buffered saline-protein solution for flow cytometric analysis of VEGF binding. This assay quantitatively determines the percentage of osteoblasts expressing biologically functional VEGF receptors within a population and estimates the receptor density for VEGF on cell surfaces.

#### Bone Nodule Formation

Human Osteoblasts were seeded in 6-well plates at a density of 1 × 105 cells/mL and cultured as described above. Upon confluence (48–72 hours after plating), 50 ug/mL ascorbic acid was added to the cultures. The cell cultures were then supplemented with recombinant human VEGF 165 (0 – 50 ng/mL), PlGF (0 – 100 ng/mL) or a monoclonal mouse anti-human VEGF neutralizing antibody (0.3 ug/mL). The treated medium was replenished daily. Mineralised nodules began to appear by 3–8 days at which time the medium was further supplemented with [beta]- glycerol phosphate and ascorbic acid to further stimulate osteogenic differentiation. After 18 days in culture, the number of mineralized bone nodules was quantified by von Kossa staining. All cultures were performed in triplicate and six fields per culture well were counted.

#### Alkaline Phosphatase Assay

Alkaline phosphatase activity in the osteoblast culture system was determined by measuring cell supernatant hydrolysis of p-nitrophenyl phosphate, yielding p-nitrophenol, which when alkaline is converted to a yellow complex easily measured by spectophotometric analysis at 400–420 nm (Sigma Diagnostics).

#### Annexin V- Fluorescein Isothiocyanate Labeling

Primary human osteoblasts were plated into six well dishes in serum free culture medium and allowed to become adherent for 8 hours. Cell cultures were then treated for 6 hours with TNFα (0 – 1000 ng/mL) in the presence and absence of anti Fas IgM (1000 ng/mL) or an isotype control IgM. These experiments were repeated in the presence of VEGF (0 – 1000 ng/mL), PlGF (0 – 100 ng/mL), bFGF (0 – 100 ng/mL), IGF-1 (0 – 200 ng/mL) or PDGF (0 – 100 ng/mL). Following trypsinization and washing (with annexin buffer, 10 mM HEPES and 0.5% bovine serum albumin), 2 × 10^5 ^cells/100 μL annexin buffer were incubated with 25 μg/mL of fluorescein isothiocyanate-labeled annexin V. Cells were then incubated at 4°C for 60 minutes, washed with and resuspended in annexin buffer and analyzed by flow cytometry. The percentage of cells staining positive for annexin V was determined using Cell Quest software (Becton Dickinson).

#### Quantification of DNA fragmentation

Primary human osteoblasts were plated into six well dishes in serum free culture medium and allowed to become adherent for 8 hours. Cell cultures were then treated for 6 hours with TNFα (0 – 1000 ng/mL) in the presence and absence of anti Fas IgM (1000 ng/mL) or an isotype control IgM. These experiments were repeated in the presence of VEGF (0 – 1000 ng/mL), PlGF (0 – 100 ng/mL), bFGF (0 – 100 ng/mL), IGF-1 (0 – 200 ng/mL) or PDGF (0 – 100 ng/mL). Following trypsinization and washing, 2 × 10^6 ^cells were gently resuspended in 1.0 mLs of hypotonic fluorochrome solution (50 μg/mL propidium iodide (PI), 3.4 mM sodium citrate, 1.0 mM Tris, 0.1 mM ethyelenediamine tetraacetic acid, 0.1% Triton X-100), and incubated in the dark at 4°C for 2 hours before they were analyzed by a FACScan flow cytometer (Becton Dickinson). The forward scatter and side scatter of cell particles were simultaneously measured. The PI fluorescence of individual nuclei with an acquisition of FL2 was plotted against forward scatter, and the data was registered on a logarithmic scale. The minimum number of 5,000 events were collected and analysed using Cell Quest software. Apoptotic cell nuclei were distinguished by their hypodiploid DNA content from the diploid content of normal cell nuclei. Cell debris was excluded from analysis by raising the forward threshold. All measurements were performed under the same instrument settings.

#### Western Immunoblotting Analysis for BCl2 Protein

Primary human osteoblasts were plated into six well dishes in serum free culture medium and allowed to become adherent for 8 hours. Cell cultures were then treated for 6 hours with TNFα (0 – 1000 ng/mL) in the presence and absence of anti Fas IgM (1000 ng/mL) or an isotype control IgM. These experiments were repeated in the presence of VEGF (0 – 1000 ng/mL) or PlGF (0 – 100 ng/mL). Following trypsinization the cells were harvested by centrifugation, washed twice in PBS and resuspended as 6 × 10^6 ^cells in 2 mLs of lysis buffer (50 mM Tris, pH 7.4, 5 mM EDTA, 250 mM NaCl, 50 mM NaF, 0.1% Triton X-100, 10 μg/mL leupeptin, and PMSF). Cell lysis was achieved after 10 minutes on ice. Protein concentrations were measured by the Bradford assay and normalized to 50 μg/lane on 12.5% SDS-polyacrylamide gel. An internal control, beta actin, was utilized to ensure that the loading quantity of protein was equal in all lanes. The gel was blotted for 150 minutes at 300 mA onto a Hybond-ECL nitrocellulose filter. The filter was washed twice with Tris buffered saline containing 0.1% Tween-20, and then nonspecific binding sites were blocked by incubation, under constant agitation, in 5% bovine serum albumin/Tris buffered saline/0.1% Tween-20 for one hour at room temperature. The filter was then incubated, under constant agitation, for two hours at room temperature with the specific rabbit anti-human polyclonal antibody to BCl2 (1:500 dilution) diluted in 3% BSA/TBS-Tween-20. The nitrocellulose filter was washed twice and detection performed for 2 hours at room temperature using horseradish peroxidaze-conjugated goat antirabbit (1:10,000 dilution) secondary antibody.

### Statistical Analysis

The following data represents the mean +/- standard error of the mean (s.e.m) in all cases. All determinations were performed in triplicate, and n = 6 experiments in each case. Single factor analysis of variance (ANOVA) was performed to determine statistical significance, and a p value < 0.05, or a confidence interval of 95% was considered significant.

## Results

### Primary Human Osteoblasts express VEGF receptors

**Figure 1:**
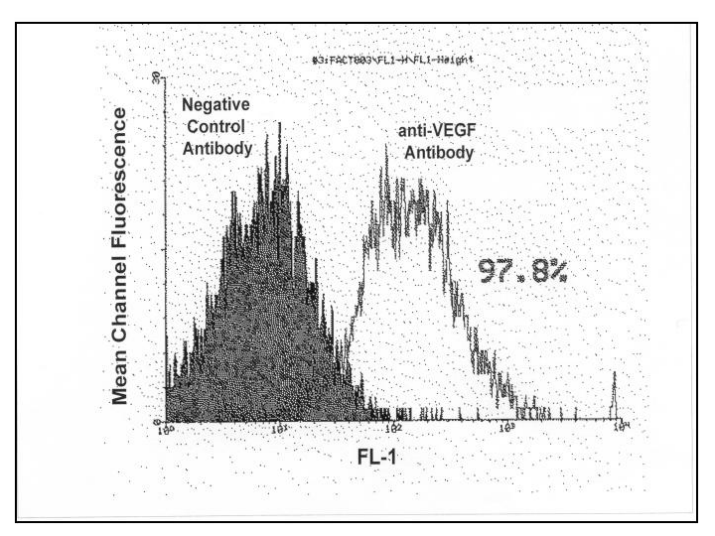
**Expression of functional VEGF receptors on Primary Human Osteoblasts**. Mean Channel Fluorescence is measured using flow cytometric analysis of avidin- FITC labelling of osteoblast rich cultures treated with a biotinylated VEGF or negative control antibody for 2 hours. The flow cytometric image shown is representative in each case. Receptor expression was performed in six separate experiments, with triplicates in each experiment.

(see Figure 1) Osteoblast rich cultures from trabecular bone explants demonstrated no significant differences in activity, receptor expression, cytokine release or response to angiogenic cytokines from the commercially available human osteoblast cell line. As shown, 97.8% of cells in the culture system expressed VEGF receptors. This biotinylated VEGF binding assay does not distinguish the VEGF receptor isotypes involved but rather indicates the functional biological activity of VEGF receptor expression in the cell population.

### Exogenous VEGF induces alkaline phosphatase release (Figure 2)

**Figure 2:**
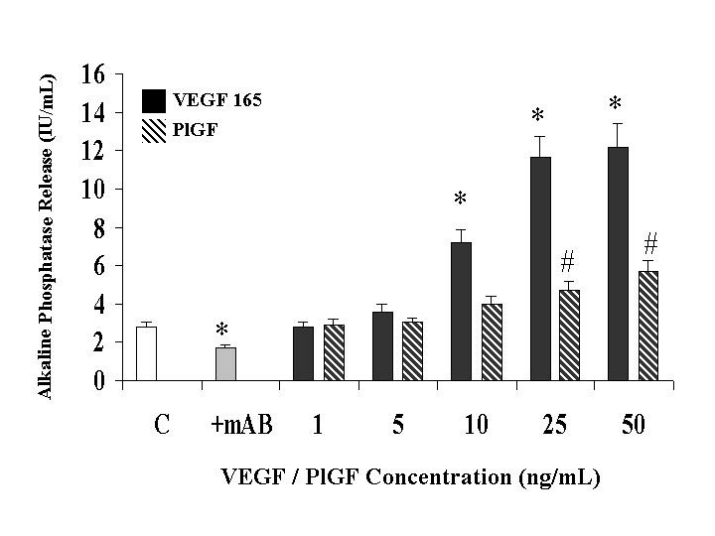
**The effect of a neutralizing monoclonal antibody and of VEGF receptor-specific ligands on Primary Human Osteoblast alkaline phosphatase release in vitro**. Bone nodule formation was assessed by von Kossa staining and alkaline phosphatase release by p-nitrophenyl phosphate hydrolysis as described in Materials and Methods. mAB: neutralizing monoclonal antibody to VEGF 165 (0.3 ug/mL), VEGF 165: vascular endothelial growth factor isotype 165, PlGF: placental like growth factor. Data illustrates mean +/- standard error of the mean in each case. The results were derived from six separate experiments, with triplicates performed in each experiment. # p < 0.05 represents statistically significant differences compared to control, * p < 0.05 represents statistically significant differences compared to VEGF 165.

The concentration of endogenously produced VEGF was measured in the cultures. The range was 0.8 – 2.25 ng/mL following 72 hours of incubation. This was within the range of exogenously administered VEGF that produced equivalent results. After 48 hours in culture, recombinant human VEGF 165 increased alkaline phosphatase release in a dose dependant manner. VEGF concentrations of 5, 10 and 25 ng/mL were sufficient to increase nodule formation 1.3-(not significant), 2.6 and 4.1-fold, over that of cultures replete of exogenous VEGF. Daily administration of 0.3 ug/mL of mAB VEGF again resulted in a significant decrease (39% reduction) in nodule formation in the cultures replete of exogenous VEGF, again highlighting the importance of this positive feedback loop. PlGF was slightly more efficacious at 25 ng/mL (66% increase) and 50 ng/mL (103% increase) in its effects on alkaline phosphatase release. This data suggests that ligation and activation of the specific VEGF receptor types has differential effects on its various mitogenic activities.

### Exogenous VEGF induces bone nodule formation in primary human osteoblasts (Figure 3)

**Figure 3:**
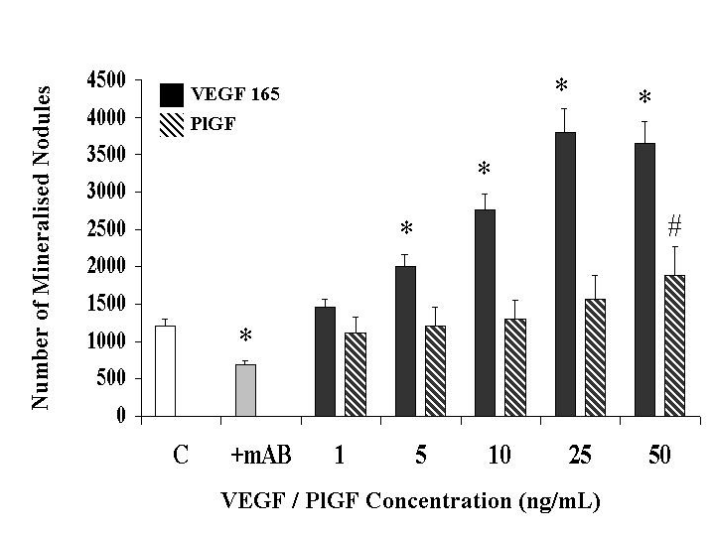
**The effect of a neutralizing monoclonal antibody and of VEGF receptor-specific ligands on Primary Human Osteoblast Bone Nodule Formation in vitro**. Bone nodule formation was assessed by von Kossa staining and alkaline phosphatase release by p-nitrophenyl phosphate hydrolysis as described in Materials and Methods. mAB: neutralizing monoclonal antibody to VEGF 165 (0.3 ug/mL), VEGF 165: vascular endothelial growth factor isotype 165, PlGF: placental like growth factor. Data illustrates mean +/- standard error of the mean in each case. The results were derived from six separate experiments, with triplicates performed in each experiment. ^# ^p < 0.05 represents statistically significant differences compared to control, * p < 0.05 represents statistically significant differences compared to VEGF 165.

After 18 days in culture, recombinant human VEGF 165 increased mineralized nodule formation in a dose dependant manner. VEGF concentrations of 5, 10 and 25 ng/mL were sufficient to increase nodule formation 1.6-, 2.3- and 3.16-fold respectively, over that of cultures replete of exogenous VEGF. Daily administration of 0.3 ug/mL of a neutralising antibody to VEGF (mAB VEGF) resulted in a significant decrease (44% reduction) in nodule formation in the cultures replete of exogenous VEGF. Addition of a control monoclonal antibody had no effect on the culture system. This data demonstrates that endogenously released VEGF is involved in a positive feedback loop that serves to stimulate osteoblast bone formation When compared to VEGF 165, Placental Growth Factor (PlGF) had only a minimal effect on mineralised nodule formation at 25 ng/mL (30% increase) and 50 ng/mL (57% increase) concentrations. This demonstrates that the Flt-1 receptor plays little role in the effects of VEGF on osteoblast formation of mineralised nodules..

### The effect of angiogenic cytokines on osteoblast apoptosis (Figure 4)

**Figure 4:**
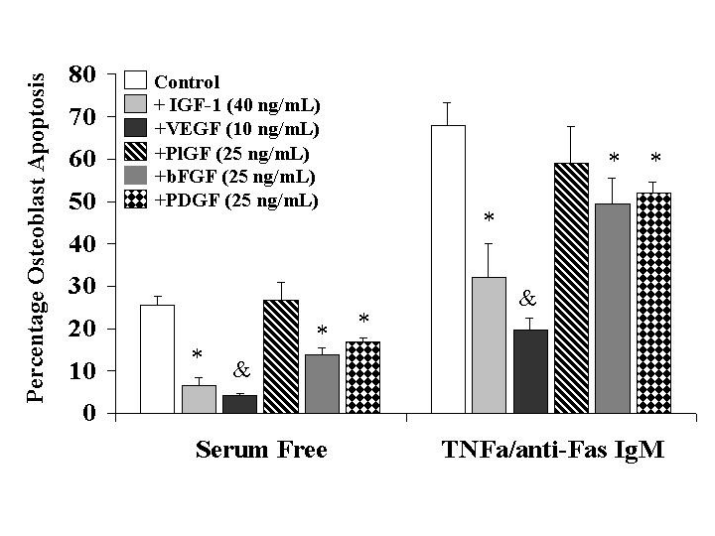
**The effects of osteotropic and angiogenic cytokines on Primary Human Osteoblast apoptosis in vitro**. Osteoblast apoptosis was determined by Annexin V- Fluorescein Isothiocyanate labelling and hypodiploid DNA measurement as described in Materials and Methods. IGF-1: insulin like growth factor -1, VEGF 165: vascular endothelial growth factor isotype 165, PlGF: placental like growth factor, bFGF: basic fibroblast growth factor, PDGF: platelet derived growth factor. Data illustrates mean +/- standard error of the mean in each case. The results were derived from six separate experiments, with triplicates performed in each experiment. * p < 0.05 represents statistically significant differences compared to control, ^&^p < 0.05 represents statistically significant differences compared to IGF-1, PlGF, bFGF and PDGF.

For this series of experiments serum free conditions were used in order to examine the effects of the various angiogenic cytokines in isolation. Control osteoblast apoptosis under these conditions was 25.6%, as compared to 3% in the presence of serum (see Figure 4). Cell cultures were treated with anti Fas-IgM (1000 ng/mL) and TNF alpha (100 ng/mL) to simulate 'pathological' apoptosis in conditions of excessive bone loss. This resulted in reproducible rates of programmed cell death of 68%. We found that TNF alpha, in the absence of Fas IgM, did not induce apoptosis of primary human osteoblasts but rather served to increase expression of Fas receptor. Thus increasing concentrations of TNF alpha resulted in increased rates of 'pathological' apoptosis (Data not shown here). Addition of 40 ng/mL of IGF-1 inhibited spontaneous and pathological apoptosis by 73.6% and 53% respectively. Treatment of the cultures with 10 ng/mL of VEGF inhibited spontaneous and TNF/Fas induced programmed cell death by 83.6% and 71% respectively. Thus VEGF afforded significantly better protection from apoptosis under both normal and particularly pathological conditions than the 'gold standard' osteotropic cytokine IGF-1. PlGF had no significant effect on the rate of osteoblast apoptosis demonstrating that the Flt-1 receptor was not involved in the survival activity of VEGF. Both bFGF (46% reduction in spontaneous and 27% reduction in pathological apoptosis) and PDGF (34% reduction in spontaneous and 23.5% reduction in pathological apoptosis) at 25 ng/mL attenuated osteoblast apoptosis, but to a significantly lesser extent. These concentrations of VEGF, bFGF and PDGF were used, as they were found to be comparable in induction of endothelial cell proliferation (an in vitro measure of angiogenesis) in preliminary studies.

### Endogenous VEGF and BCl2 regulate osteoblast apoptosis. (Figure 5)

**Figure 5:**
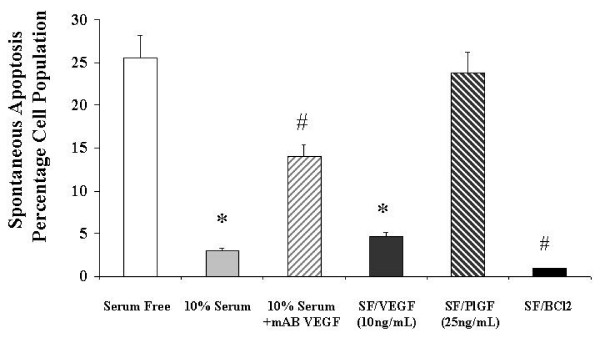
**The effects of 10% serum, a neutralizing monoclonal antibody and of VEGF receptor-specific ligands on spontaneous Primary Human Osteoblast apoptosis in vitro**. Osteoblast apoptosis was determined by Annexin V- Fluorescein Isothiocyanate Labeling and hypodiploid DNA measurement as described in Materials and Methods. mAB VEGF: neutralising monoclonal antibody to vascular endothelial growth factor isotype 165 (0.3 ug/mL), VEGF: vascular endothelial growth factor, PlGF: placental like growth factor. Data illustrates mean +/- standard error of the mean in each case. The results were derived from six separate experiments, with triplicates performed in each experiment. * p < 0.05 represents statistically significant differences compared to control, ^# ^p < 0.05 represents statistically significant differences compared to 10% serum alone.

The percentage of cells staining positive for annexin V was analyzed by flow cytometry. Spontaneous apoptosis at 24 hours in the absence of serum is 25.6%. Pretreatment of the osteoblasts with VEGF 10 ng/mL (4.7% apoptosis rate) was almost as effective as culture in the presence of 10% serum (3% apoptosis rate) in inhibiting spontaneous programmed cell death.. Pretreatment of the cultures with a neutralising monoclonal antibody to VEGF (mAB VEGF), in the absence of exogenous VEGF resulted in a spontaneous apoptosis rate of 14%. This indicates that VEGF released in culture by primary human osteoblasts is integral to the regulation of the rate of programmed cell death. PlGF had no effect on apoptosis (23.8%), again demonstrating that the Flt-1 receptor was not involved in the survival activity of VEGF.

### VEGF attenuates osteoblast apoptosis by enhancing expression of BCl2 gene (Figures 6 and 7)

**Figure 6:**
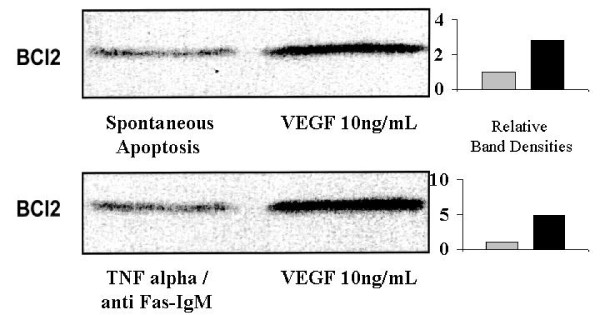
**The effect of exogenous VEGF 165 on primary human osteoblast BCl2 expression during spontaneous and 'pathological' apoptosis in vitro**. Expression of the antiapoptotic gene BCl2 was determined using Western immunoblotting as described in Materials and Methods. Spontaneous apoptosis was measured for cells in serum free conditions. Pathological apoptosis was induced by treatment of the cultures with TNFa and anti-Fas IgM. BCl2: antiapoptotic gene, VEGF: vascular endothelial growth factor 165, TNFa: tumour necrosis factor alpha (100 ng/mL), anti-Fas IgM: anti Fas receptor immunoglobulin (1000 ng/mL). The immunoblot shown is representative in each case. Western Immunoblotting was performed for six separate experiments, with triplicates in each experiment.

**Figure 7:**
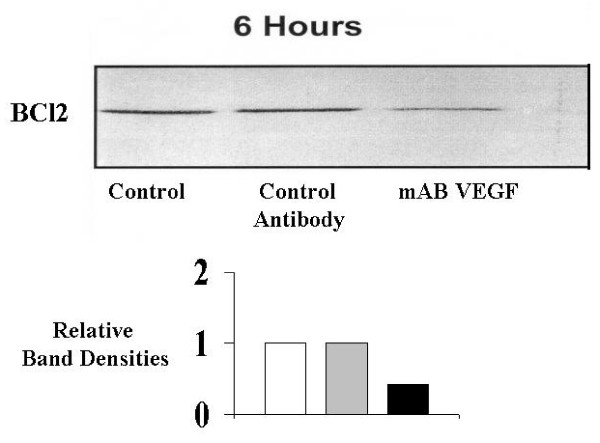
**The effect of neutralisation of endogenous VEGF on primary human osteoblast BCl2 expression during spontaneous apoptosis in vitro**. Expression of the antiapoptotic gene BCl2 was determined using Western immunoblotting as described in Materials and Methods. Spontaneous apoptosis was measured for cells in serum free conditions. BCl2: antiapoptotic gene, mAB VEGF: neutralising monoclonal antibody to vascular endothelial growth factor isotype 165 (0.3 ug/mL), control antibody: isotype control antibody with no biological activity in vitro. The immunoblot shown is representative in each case. Western Immunoblotting was performed for six separate experiments, with triplicates in each experiment.

Western Immunoblotting confirms that pretreatment of the osteoblast cultures with exogenous VEGF 10 ng/mL, results in up-regulation in expression of the anti-apoptotic gene BCl2, reflecting a decrease in the rates of programmed cell death (Figure 5). This is true for both spontaneous and pathological (TNF alpha/anti Fas-IgM induced) apoptosis, with relative band density increases of 4.9 and 2.8 respectively. Treatment of the cultures with a neutralising monoclonal antibody to VEGF 0.3 ug/mL (mAB VEGF), in the absence of any exogenous VEGF, resulted in a downregulation of BCl2 expression with relative band density decreases of 0.43 and 0.31 for spontaneous and pathological apoptosis respecively. These decreases in anti-apoptotic gene expression reflect the increased apoptotic rates of these cultures as seen in Figure 4. These data demonstrate that the significant protective effect of VEGF on primary human osteoblasts is mediated by expression of the critical antiapoptotic gene BCl2.

## Discussion

Bone formation and resorption is the function of the basic multicellular unit (BMU), where osteoblasts and osteoclasts interact with one another and with haemopoietic and stromal cells of the bone marrow [1]. Regulation of the numbers and activities of bone cells is essential for skeletal homeostasis while mismatch between formation and resorption is largely responsible for most systemic and localised bone diseases. The rate of bone formation is largely determined by the number of osteoblasts, which in turn is determined by the rate of replication of progenitors and the life-span of the mature cells, reflecting timing of cell death by apoptosis [2]. Current evidence suggests that apoptosis is the fate of the majority of osteoblasts, and that changes in the prevalence of osteoblast apoptosis alter the balance of skeletal homeostasis [1,2,23,25-29]. Glucocorticoid induced osteopenia is characterised by increased osteoblast apoptosis, a phenomenon which is reversible by estrogen administration in vitro and in vivo [30]. Bisphosphanates and parathyroid hormone increase bone formation by prevention of osteoblast apoptosis, suggesting novel therapeutic strategies for osteoporsis. Active and coordinated apoptosis of cells within the post traumatic bone callus is thought to regulate local release of cellular agents that modulate fracture repair [13,24]. Disuse osteopenia, periprosthetic and infection related bone loss are all associated with increases in 'pathological' osteoblast apoptosis, enhanced cell suicide mediated by proinflammatory cytokines such as TNF alpha, anti CD 95-IgM, Il-1β and Il-6 [2,17-20,22]. The vasculature of bone is an essential component of bone repair, remodeling and growth, however the precise interactions between vascular cells and bone forming cells are still unclear [28]. The bone loss of osteoporosis is characterised by reduced capillary compartments, distraction osteogenesis increases bone formation with a parallel increase in capillary number and vascularised bone grafts are far superior to conventional ones. Bone chamber models using intravital microscopy and microangiographic studies have demonstrated that neovascularization temporally precedes neo-osteogenesis [28]. Endothelial cells cocultured with fetal rat calvaria induce bone formation. Following bone injury the developing osteoprogenitor cells and osteoblasts are in intimate contact with the basement membrane of the invading capillaries. During fracture healing ossification by osteoblasts is spatially associated with sites of capillary penetration into the callus [8]. Normal remodeling of mature bone occurs in discrete vascularised elongated structures, termed osteons, in which old bone is resorbed by osteoclasts at the cutting cone, and the defect is filled with new bone by trailing osteoblasts. During skeletogenesis vascular endothelial growth factor (VEGF) mediated blood vessel invasion of the growth plate coincides with mineralisation of the extracellular matrix (ECM), apoptosis of hypertrophic chondrocytes and bone formation [9]. We have previously reported that musculoskeletal injury results in systemic and fracture site angiogenic responses in the human, that are primarily mediated by vascular endothelial growth factor (VEGF) [6,7]. We have also demonstrated that VEGF is essential for both intramembranous and endochondral bone formation, and exogenous enhances fracture repain in a number of animal models [8]. As outlined earlier, osteoblasts release VEGF in the setting of bone injury. They express receptors for VEGF in a differentiation dependent manner [4], while osteoclasts express VEGF receptors and osteoclast differentiation and bone resorption is enhanced by VEGF [16]. Thus VEGF may represent a principal regulator of the activities of the BMU, under normal and indeed pathological conditions. The aim of this study was to determine the role of VEGF on the activity and life span of primary human osteoblasts in vitro.

We utilised both osteoblast rich cultures from human trabecular bone explants and commercially available primary human osteoblasts in this study. We could not determine any significant differences in activity, receptor expression or patterns of apoptosis between the two populations. Previous studies have examined some of the features of osteoblast apoptosis, but these have used transformed cell lines e.g. murine MC3T3-E1 and human MG-63 [13,14,29]. As these cell lines have been immortalised accurate conclusions cannot be drawn based on their responses to pro and/or antiapoptotic stimuli. It has previously been reported that the murine pre-osteoblast cell line KS483 express VEGF receptor isotypes in a differentiation dependant manner [4]. In order to examine the effects of VEGF on primary human osteoblasts we first demonstrated that these cells express functionally active VEGF receptors. Using a biotinylated VEGF binding assay we were able to show that 97.8% of the osteoblast-rich cell population expressed VEGF receptors. Thus we could anticipate a response of these cells to exogenously administered VEGF to the culture system. Performing parallel experiments with Placental Growth Factor allowed us to examine the relative roles of KDR and Flt-1 receptors in the mitogenic and antiapoptotic effects of VEGF. As PlGF has high affinity for Flt-1 and does not bind KDR, it's effects reflect specific activation of the Flt-1 receptor. Differences in the effects of VEGF 165 and PlGF, at comparable concentrations, can therefore be attributed to activation of KDR or perhaps neuropillin receptors. Using a variety of VEGF selective mutants Gerber et al have reported that the antiapoptotic effects of VEGF on endothelial cells are mediated primarily by KDR receptor through the activation of P-13 kinase [5]. VEGF mRNA expression in osteoblasts is increased by several factors e.g. PGE 1 and 2, IGF-1Vit D3, TGFβ and hypoxia [10,12,13,15,30]. However, to date it was not known if VEGF had a direct effect on osteoblasts themselves. Our present data demonstrates that primary human osteoblasts are stimulated by exogenously administered VEGF 165 to increase mineralised nodule formation and alkaline phosphatase release. Therefore VEGF has direct osteotropic effects independant of a prevailing vasculature. The concentrations of VEGF that were used in this experiment are similar to those measured in plasma of patients with isolated long bone fractures, those relesased by stimulated osteoblasts in vitro, and to those shown to enhance osteoclastic bone resorption and survival of mature osteoclasts. In that report using purified mature rabbit osteoclasts the specific VEGF receptor isoform involved was not examined [16]. Our data clearly demonstrates that the mitogenic effects of VEGF on human osteoblasts are not mediated by the flt-1 receptor and so are mediated by either KDR or neuropillin. The results of treatment with PlGF show that Flt-1 has little or no role in mediating mineralisation but some limited role in activation of alkaline posphatase release. While this appears contradictory, it must be remembered that the process of mineralisation is far more involved than just release of a single protein. KDR activation on the osteoblast appears to signal all the coordinated processes required to lay down bone, while Flt-1 activation does not achieve this level of cell mitogenesis. Neutralisation of endogenous VEGF in our cell culture system had a significant effect on mineralisation and alkaline phosphatase release. Thus osteoblasts release VEGF in an autocrine fashion regulating their own activity. These data taken together, it is likely that VEGF can mediate either bone formation or resorption, the ultimate balance depending on cell receptor expression, differentiation state, and the cytokine, biophysical and biochemical milieu of the basic multicellular unit. Perhaps this may explain why the degree and nature of vascularisation must be optimal for the formation of bone. Osteonecrosis is characterised by relative avascularity, failure to reestablish appropriate blood supply signals atrophic fracture non-union, while excessive bone resorption is associated with large, disorganised vascular channels. Systemic disorders such as polyostotic Pagets disease, with simultaneous pathological bone formation and resorption, may well represent a critical breakdown in the coupling of skeletal homeostasis and angiogenesis as mediated by various factors including VEGF.

Abnormalities in cell death control contribute to a variety of diseases including cancer, autoimmunity, degenerative disorders and osteoporosis [1,2,17,23]. Signaling for apoptosis occurs through multiple independent pathways that are initiated either from triggering events within the cell e.g. mitochondrial membrane depolarisation or from outside the cell, e.g. ligation of the death receptors [25,26]. The ligation of the Fas or CD 95 receptor by its ligand initiates programmed cell death in a number of normal cell types [31-33]. This mechanism is implicated as the principle pathway for excessive apoptosis in many pathological conditions. Ligation of Fas with the Fas receptor activates an intracellular cascade of cysteine proteases (caspases) that ultimately dismantles the cell and facilitates phagocytosis by neighboring cells. While caspase 3 is the common terminal protease to all pathways of apoptosis, caspase 6 and 8 are known to specifically signal the activation of the death receptor pathway. Our data herein demonstrates that TNF alpha of itself does not induce apoptosis of primary human osteoblasts but rather increases the expression of the death receptor Fas. Tsuboi et al have previously reported comparable data for the human osteoblast cell line MG63 [33]. Thus in the presence of Fas ligand, as in inflammatory and septic conditions of bone, high levels of TNF alpha may contribute to the associated osteolysis. Treatment of the cells with IETD-FMK, a specific inhibitor of caspases 6 and 8 completely reverses the effect of Fas receptor activation, confirming the specificity of this pathway in pathological apoptosis of primary human osteoblasts. Using mouse calvarial osteoblasts Hill et al demonstrated that IGF-1 was the most potent inhibitor of apoptoss of 20 growth factors tested, however, not including VEGF [19]

Our study demonstrates that VEGF released in culture by primary human osteoblasts is integral to the regulation of the rate of programmed cell death. PlGF had no effect on apoptosis demonstrating that the Flt-1 receptor was not involved in the survival activity of VEGF. Transfection of the osteoblasts with the antiapoptotic gene BCl2 was sufficient to inhibit osteoblast apoptosis induced by serum starvation, demonstrating that BCl2 levels are critical for regulation of osteoblast life-span.

Western Immunoblotting confirmed that pretreatment of the osteoblast cultures with exogenous VEGF resulted in up-regulation in expression of the anti-apoptotic gene

BCl2 and a decrease in the rates of programmed cell death. Treatment of the cultures with a neutralising monoclonal antibody to VEGF, in the absence of any exogenous VEGF, resulted in a downregulation of BCl2 expression and a parallel increase in apoptotic rates of these cultures. These data demonstrate that the significant protective effect of VEGF on primary human osteoblasts is mediated by expression of the critical antiapoptotic gene BCl2.

In conclusion, our data demonstrates that VEGF is a potent regulator of osteoblast life-span in vitro, attenuating both spontaneous and pathological programmed cell death. This autocrine feedback mechanism is critical to the survival of these cells and is mediated primarily via non flt-1 receptor mediation and expression of BCl2 antiapoptotic gene.

## Competing interests

The authors declare that they have no competing interests.

## Authors' contributions

Both authors, JS and BL were involved in the design and execution of the experimental studies described in this manuscript. Both authors are responsible for writing and editing the manuscript. Both authors have read and approved the final manuscript for publication.
